# An educational intervention to promote a culture of gender equity among persons with traumatic brain injury and caregivers: A pilot study

**DOI:** 10.3389/fresc.2023.1160850

**Published:** 2023-04-26

**Authors:** Sara Hanafy, Enrico Quilico, Halina (Lin) Haag, Yuelee (Ben) Khoo, Sarah Munce, Sally Lindsay, Angela Colantonio, Tatyana Mollayeva

**Affiliations:** ^1^ Rehabilitation Sciences Institute, University of Toronto, Toronto, ON, Canada; ^2^ KITE-Toronto Rehabilitation Institute, University Health Network, Toronto, ON, Canada; ^3^ Bloorview Research Institute, Holland Bloorview Kids Rehabilitation Hospital, Toronto, ON, Canada; ^4^ Faculty of Social Work, Wilfrid Laurier University, Waterloo, ON, Canada; ^5^ Dalla Lana School of Public Health, University of Toronto, Toronto, ON, Canada; ^6^ Department of Occupational Science and Occupational Therapy, University ofToronto, Toronto, ON, Canada; ^7^ Institute of Health Policy, Management and Evaluation, University of Toronto, Toronto, ON, Canada

**Keywords:** biological sex, brain injuries, concussion, education, gender role, knowledge acquisition, rehabilitation

## Abstract

**Background:**

Traumatic brain injury (TBI) outcomes are dependent on patients' biological sex (e.g., hormone levels) and sociocultural gender (e.g., norms, responsibilities). Informal caregivers additionally experience disruptions to identity and roles post-TBI. However, information on this topic remains largely unavailable to patients and caregivers.

**Purpose:**

This study aimed to determine the effectiveness of a one-time educational intervention on sex and gender influences in TBI for patients and informal caregivers.

**Materials and methods:**

We conducted a pilot pre-test/post-test randomized control-group design study. Groups (i.e., passive, active and control) consisted a total of 16 persons with TBI and caregivers (75% persons with TBI, 63% women). Individual and group learning gains, and group-average normalized gain, were computed for three learning domains: knowledge, attitude, and skill. An intervention with an average normalized gain of ≥30% was considered effective. Educational intervention evaluation and qualitative comments post-participation were summarized.

**Results:**

The passive group demonstrated the highest average normalized gain across the three learning domains, including 100% for knowledge, 40% and 61% for attitude, and 37% for skill. The remaining groups did not reach an average normalized gain of ≥30%, except for the attitude domain of the control group (33% and 32%). Two key categories were identified qualitatively: (1) gendered self-expectations post-injury and (2) implications of gender stereotypes in rehabilitation, including the need for rehabilitation treatment to look beyond sex and gender. The post-participation educational session evaluation conveyed high appraisal of content, organization, and usability of the intervention.

**Conclusion:**

A one-time passive educational intervention on sex and gender in TBI may improve knowledge, attitude, and skill on the topic of sex and gender among adults with TBI and caregivers. Obtaining knowledge and skill on sex and gender effects in TBI can potentially help persons with TBI and caregivers adapt to changes in roles and behaviours post-injury.

## Introduction

Traumatic brain injury (TBI) is among the most disabling of injuries affecting many individuals in the prime of their lives (1). It is burdensome to patients (2), and is associated with challenges to community integration and compromised self-care requiring ongoing support of caregivers (3, 4). An individual's physiological sex and/or sociocultural gender can significantly influence the course of TBI making sex and gender effects essential to consider in rehabilitation and care (5). Sex refers to the biological features in humans (6), while gender refers to the socially constructed roles, behaviours, expressions, and identities of men, women, and gender-diverse people (6).

A recent systematic review supports sex and gender differences in TBI clinical and functional outcome, related to sex effects and gender inequities (7). Some of these differences help to explain why women with concussions are at a greater risk of post-concussive symptoms (8, 9) and experience less favourable functional outcome in mild and moderate injuries (10–12), and why men tend to fare worse in executive functioning following moderate-to-severe injuries (12, 13). Although the variability in recovery time and outcomes between men and women with TBI were thought to predominantly reflect biological differences through sex hormone levels (11, 14–16), recent research suggests gender-based division of labour and access to financial resources as additional meaningful indicators (12, 17). Gender further plays a role post-TBI through the experienced loss of masculine and feminine identities and disruptions in gender-specific roles and relationships with social implications (18–20). Gender also shapes the experiences of caregivers supporting individuals with TBI, highlighting the caregivers' paralleled identity loss and adaptation to new roles and responsibilities (21–24). Based on this evidence, accounting for the gendered dynamics between people with TBI and their caregivers and considering how shifts in gender roles can influence recovery and outcomes are timely.

Equally important are the knowledge gaps and stigma that shape attitudes towards the topic of sex and gender in TBI, which affects people's understandings, behaviours, and relationships. Men and women with TBI and their caregivers are affected differently by TBI based on their sex and gender, but often are unable to explicitly acknowledge their influence on recovery post-injury (25). Depicting the gendered experiences of men and women with TBI and their caregivers may change the way people think about gender in their lives, reduce the stress and fear associated with the inability to perform normative roles, express behaviours prescribed to men and women in society, and provide care that reflects the needs of men and women with TBI (26).

Previous educational interventions have focused on the effects of TBI along with problem-solving, communication, and advocacy skills during rehabilitation for family members with TBI (27); however, addressing sex and gender inequity in TBI recovery and outcome has not been the focus in the knowledge and skill-based teachings (27). A recent scoping review on brain injury education to patients with TBI and their family members reported that the educational content provided about mild TBI varied (28). For moderate-to-severe TBI, multicomponent interventions that include education were mostly used, which masked the sole impact of the educational content (28). Further, a systematic review focusing on interventions for caregivers of TBI survivors and patient/caregiver dyads demonstrated that interventions mainly targeted support and skill-building training (29).

Laying the foundation for promoting a culture of gender equity through an education program on sex and gender effects in TBI can provide persons with TBI and their caregivers with an understanding of expectations of recovery and outcomes and with techniques to recognize harmful attitudes and behaviours by patients and caregivers. The impact of educational/knowledge transfer initiatives on patients and caregivers however has not been studied. The purpose of this pre-test/post-test comparative pilot study was to determine whether providing patients and caregivers with education about identifying and addressing sex and gender effects would alter their knowledge, attitude, and skills (30). We hypothesized that the educational intervention given to patients with TBI and caregivers would provide new knowledge, form new attitudes, and develop new skills.

## Materials and methods

### Study design

We conducted a pilot randomized control-group pre-test/post-test (31) educational intervention study at the University Health Network (UHN), Ontario, Canada. This study was part of a larger research program funded by the Canadian Institutes of Health Research (CIHR) (30). Study procedures have been approved by the UHN and the University of Toronto Research Ethics Boards. Study reporting followed the Guideline for Reporting Evidence-based practice Educational interventions and Teaching (GREET) checklist (32), based on the Template for Intervention Description and Replication (TIDieR) guidance (33).

### Study procedures

Research participants were randomly assigned into one of three groups: passive, active, or a control group. Randomization was performed using an online random team generator. Following group allocation, a pre-test questionnaire link (please refer to Development of Educational Assessment) was emailed to each participant *via* the UHN Research Electronic Data Capture (REDCap) platform (34). All study participants across groups completed the pre-test questionnaire. A one-time educational intervention was scheduled and held through Zoom in August and September of 2021 for the passive and active groups. The control group did not receive an educational intervention. The educational intervention comprised of an hour and 30-minute online session with two intermittent five-minute breaks.

Two study researchers (SH, EQ) collaboratively delivered the educational intervention to the passive and active groups. Identical content was presented in a recorded script and PowerPoint format to passive and active groups with two main differences—the active group utilized video and audio content to replace lecture-based aspects of the passive intervention [i.e., CIHR video on definitions of sex and gender (35), audio vignettes on presented case studies] and involved a live discussion among study participants for the opportunity to jointly reflect on the provided case studies.

A post-test questionnaire link was then emailed to each study participant *via* REDCap (34). All study participants across groups completed the post-test questionnaire. Study participants allocated to the educational intervention (i.e., active and passive groups) were required to complete the post-test questionnaire within 24 h of the session. The educational assessment was identical for the pre- and post-testing across the three groups. However, an educational session evaluation was included to the post-testing of the active and passive groups. All research participants received a reimbursement of $80 for their time and effort.

### Recruitment and eligibility

Study recruitment was facilitated by the UHN, the University of Toronto, and community-based brain injury organizations and included online advertisement of the study flyer through social media platforms and at national brain injury conferences. Recruitment additionally involved contacting past research participants who contributed in 2018 to the earlier phase of the research program. Individuals were eligible for inclusion if they were adults (≥18 years) with a diagnosis of a TBI and/or caregivers of individuals with a TBI. Persons with severe neurocognitive deficits or limited English literacy were excluded (30).

### Development of educational material

The intervention ‘How sex and gender affect traumatic brain injury' had three primary learning objectives for persons with TBI and caregivers: (1) to understand the difference between sex and gender constructs, (2) to understand what TBI is and its impact on recovery through a sex and gender lens, and (3) to understand how gender may affect giving and receiving care in TBI. Study author TM initially created the intervention content. The developmental process included analyses of the needs and knowledge of patients with TBI and their caregivers from semi-structured interviews (19, 20, 25) and a systematic review which identified relevant concepts based on gaps in the knowledge and needs assessment of patients with TBI and their caregivers (7). Five researchers appraised the content independently and discussed it with TM; a team discussion occurred when a lack of clarity was observed, after which consensus was reached. Two UHN patient education officers simplified the content and presentation layout to ensure both aspects met patient and caregiver education accessibility criteria. A professional design group (36) enhanced the communication and digital design of the educational material.

### Development of educational assessment

The educational assessment was created by TM based on common trends from qualitative interviews (19, 20, 25) and the systematic review findings described above (7), which were reviewed by six researchers. The educational assessment included four sections: general information (i.e., participant's sex and gender, age, and years living with TBI/supporting a person with TBI), knowledge assessment, attitude assessment, and skill assessment and can be accessed in Supplementary S1. The attitude assessment included multiple-choice (questions one and two) and Likert-scale questions (questions three to five), and was divided into two sections for analytical purposes.

### Outcome assessment

The outcome variables included individual and group gains in knowledge, attitude, and skill from pre-intervention to post-intervention. Further, the post-educational session evaluation (Supplementary S1) assessed whether the educational intervention (1) met the stated learning objectives, (2) met overall expectations, (3) was well organized, (4) was helpful and (5) contained any perceived degree of unfairness towards men, women, or gender-diverse people.

### Data analysis

#### Quantitative data

Descriptive statistics of patients with TBI and caregivers' characteristics were calculated for categorical and continuous variables. Single-patient or caregiver actual and absolute gain, and group-average relative, absolute, and normalized gain were used as objective measures of knowledge, attitude, and skill learning (37, 38).

Individual actual gain (Gi) and absolute gain (Δi) were calculated for each of the 16 study participants using the formulas below:

$$\begin{array}{rl} & \mathrm{Gi}=\mathrm{post}\text{-}\mathrm{test}\;\mathrm{score}-\mathrm{pre}\text{-}\mathrm{test}\;\mathrm{score}\\ & \Delta i=\mathrm{Gi}/\mathrm{maximum}\;\mathrm{score}\;\mathrm{achievable} \end{array}$$

Group absolute gain (Δ), group relative gain (C), and group-average normalized gain (<g>) were calculated for each group. The maximum score achievable for the calculations of individual and group absolute gains indicated the maximum number of correct responses that can be obtained in each learning domain. The group-average normalized gain is defined as the ratio of the actual average gain to the maximum possible average gain (37) and was calculated by adding individual normalized gains (<gi>) for the 16 study participants and dividing the value by the total number of individuals (38). Formulas used for the group calculations included:

$$\begin{array}{c} \;\Delta =\mathrm{average}\;\mathrm{Gi}/\mathrm{maximum}\;\mathrm{score}\;\mathrm{achievable}\;\\ \;C=\mathrm{average}\;\mathrm{Gi}/\mathrm{average}\;\mathrm{pre}\text{-}\mathrm{test}\;\mathrm{score}\;\\ <\mathrm{gi}>\;=\;[\mathrm{post}\text{-}\mathrm{test}\;\text{\%}-\mathrm{pre}\text{-}\mathrm{test}\text{\%}]/[100-\mathrm{pre}\text{-}\mathrm{test}\;\text{\%}]\; \end{array}$$

In instances where the post-test score was lower than the pre-test score, which depicted a negative gain, the values were replaced by zero (38–40).

A group-average normalized gain of ≥30% was used to determine the effectiveness of the educational intervention, as previous literature has suggested that a normalized gain of 0.30 (30%) is considered the lower margin of what would be a “medium” normalized gain (38). The measure of normalized gain is a valuable tool in analyzing pre-survey and post-survey scores to evaluate an intervention's effectiveness (41).

Quantitative data was entered and analyzed manually and *via* Excel. Figures were created using the R Project for Statistical Computing software, version 4.1.2 (42) and *pheatmap* package (43).

#### Missing data and sensitivity analysis

Data that was missing not at random (MNAR) was to be assigned a value of zero for computations. A sensitivity analysis of a simple mean imputation was to be conducted, in which missing values were to be replaced by the mean value for that variable (44). This approach ensured that a value of zero did not lead to biased effectiveness estimates.

#### Qualitative data

Open-ended questions about attitudes on gender stereotypes and their impact on recovery were summarized qualitatively, specifically through a descriptive qualitative design (45) and content analysis approach (46). Study author (SH) coded responses to these questions and generated categories with supporting quotes (46).

### Sample size

The pilot-nature of this study sought to assess whether the effectiveness of the intervention was consistent with expectations (47), and ensure the subcomponents of the study (i.e., recruitment, randomization, assessment) can be adequately executed for the future randomized controlled trial (48). A sample size of 16 has been shown to provide reliable estimates and correspond to less than 20% of underpower probability (49).

## Results

**Figure 1:**
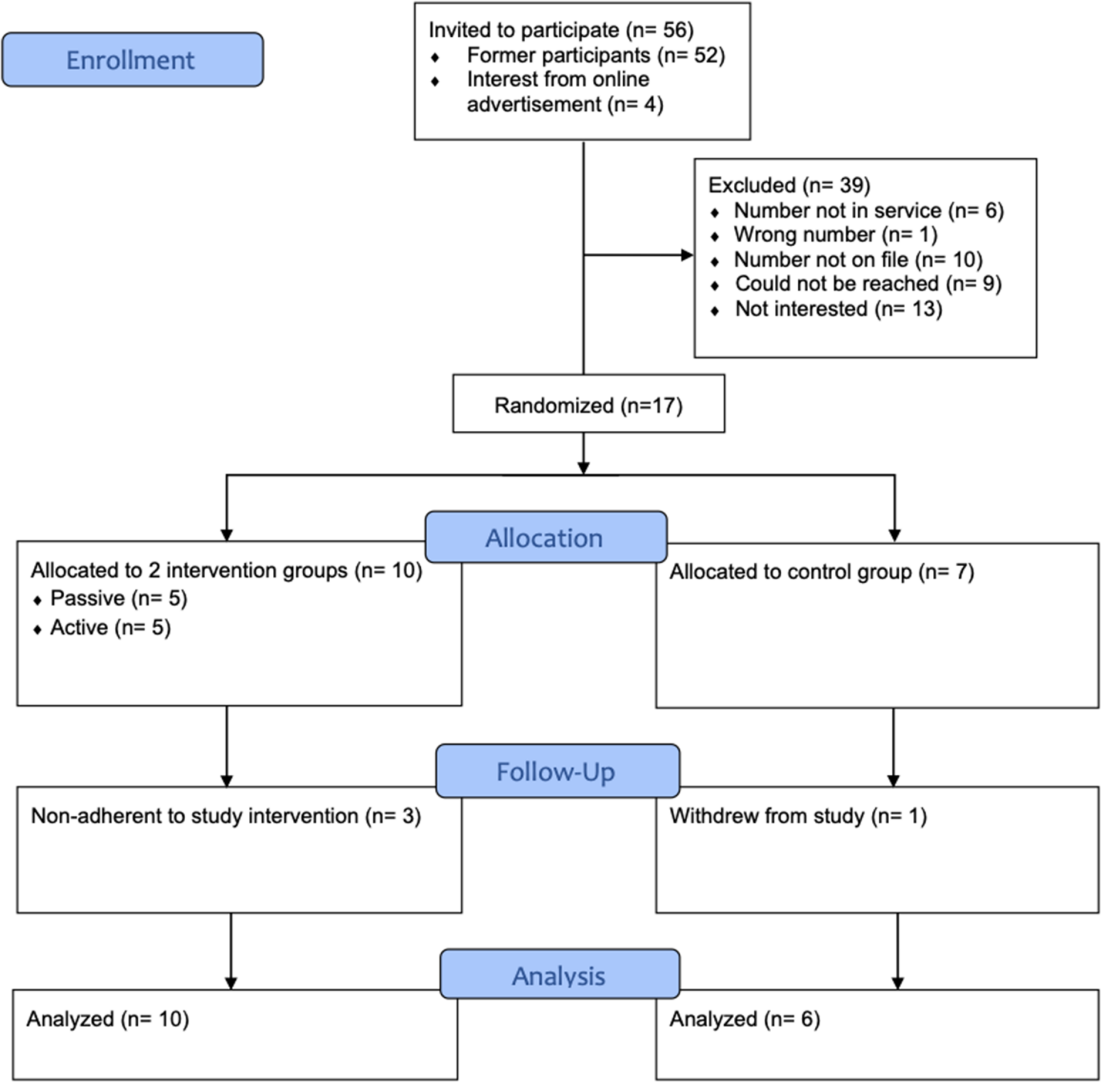
Flow diagram of participant recruitment.

A total of 17 participants (13 persons with TBI and four caregivers) provided their consent. Following randomization, five participants were allocated to the passive and active groups and seven to the control group. One participant, who was assigned to the control group, withdrew from the study decreasing the sample size to 16 research participants (Figure 1). Three individuals (one from passive and two from active group) did not attend the educational intervention; however, their responses were analyzed within the initially allocated groups, following the intention-to-treat analysis principle (50, 51).

### Sample characteristics

The final study sample consisted of 16 adults aged 27 to 79 years [median = 49, Interquartile Range (IQR) = 24.25], of which 62.5% were female. Participants included 12 persons with TBI and four informal caregivers. The time since injury/years supporting family members with TBI ranged from one to five years and five to ten years (Table 1).

**Table 1:** Demographic characteristics of the sample.

Demographics	Participants		
	Control(n = 6)	Passive(n = 5)	Active(n = 5)
Age (years), median (IQR)	60.0 (22.50)	42.0 (13.0)	42.0 (20.0)
Sex, *n* (%)			
Male	2 (33)	1 (20)	3 (60)
Female	4 (67)	4 (80)	2 (40)
Years living/supporting a person with TBI, *n* (%)			
1–5 years	4 (67)	5 (100)	4 (80)
>5–10 years	2 (33)	0 (0)	1 (20)

IQR, interquartile range; TBI, traumatic brain injury.

### Outcome assessment

#### Knowledge, attitude & skill learning gains

All 16 participants completed the pre- and post-test questionnaires. Analyses examined the median pre-test/post-test score, absolute gain, relative gain, and average normalized gain for each group and across learning domains (Table 2). With respect to knowledge, the average normalized gain was 100% for the passive group; 25% for the active group; and 25%, for the control group. With respect to attitude, the average normalized gain was 40% and 61% for the passive group; 20% and 9.2% for the active group; and 33% and 32% for the control group. With respect to skill, the average normalized gain was 37% for the passive group; 23% for the active group; and 5.5% for the control group.

**Table 2:** Pre-test/post-test scores and learning gain across three groups. Robustness of educational intervention defined if <g> is greater or equal to 30%.

Learning domain		Median Pre-test Score (IQR)	Median Post-test Score (IQR)	Absolute Gain (*Δ*)	Relative Gain (C)	Average Normalized Gain (<g>)
Passive	Knowledge	25% (25.00)	100% (0)	60%	150%	100%
	**Attitude**					
	#1,2	50% (50.00)	100% (0)	20%	29%	40%
	#3–5	73% (13.00)	87% (20.00)	12%	16%	61%
	Skill	60% (25.00)	80% (5.00)	15%	24%	37%
Active	Knowledge	75% (25.00)	50% (50.00)	10%	14%	25%
	**Attitude**					
	#1,2	100% (50.00)	100% (0)	10%	14%	20%
	#3–5	87% (20.00)	80% (40.00)	1.3%	1.5%	9.2%
	Skill	70% (20.00)	60% (30.00)	7%	8%	23%
Control	Knowledge	50% (37.50)	75% (18.75)	21%	42%	25%
	**Attitude**					
	#1,2	75% (87.50)	100% (37.50)	33%	57%	33%
	#3–5	70% (11.25)	77% (26.50)	11%	16%	32%
	Skill	63% (30.00)	53% (20.00)	3.3%	5.5%	5.5%

IQR, interquartile range.

**Figure 2:**
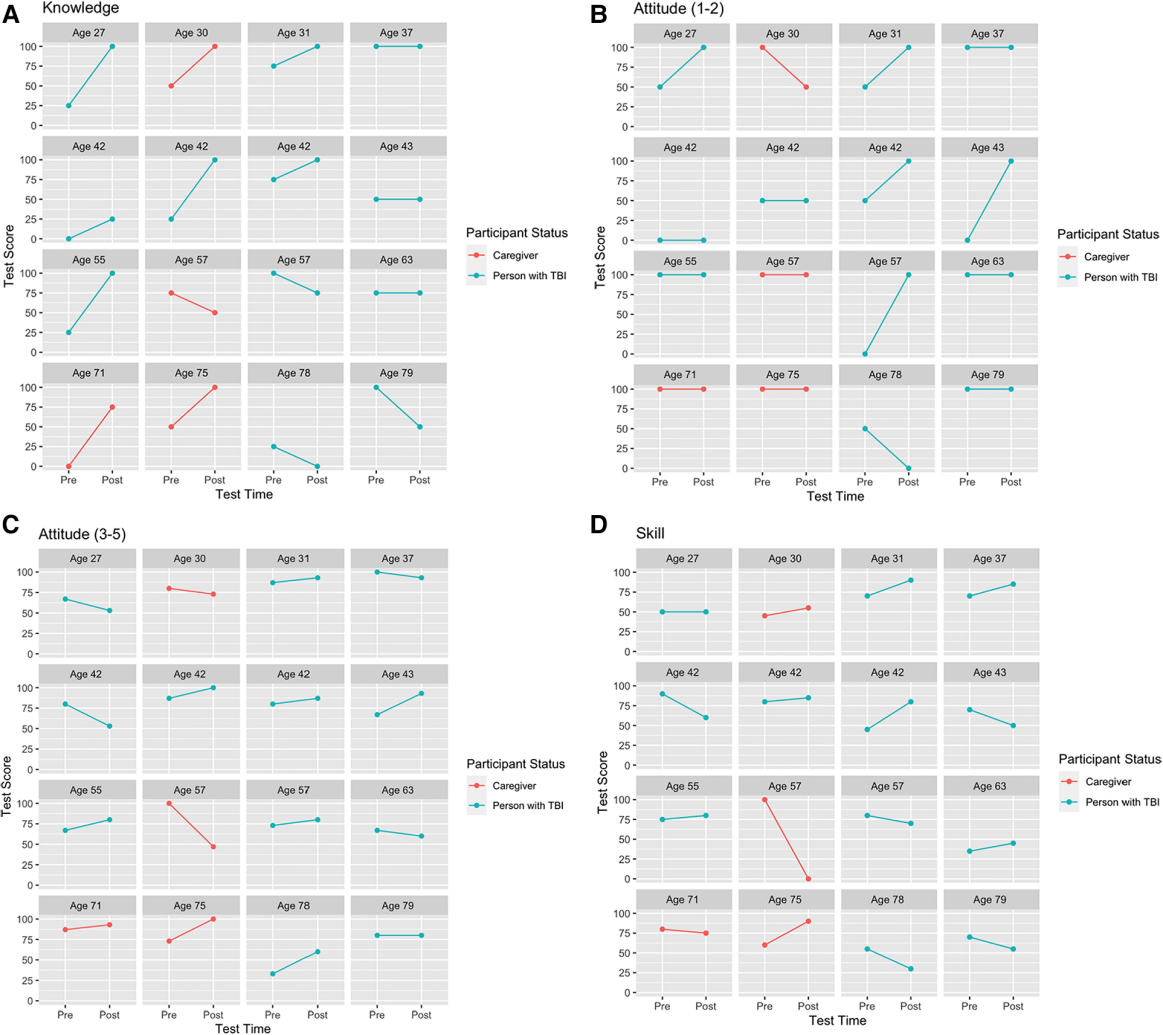
(**A–D**) by age. Learning pre- and post-test score. Plots of each person for (**A**) knowledge, (**B**) attitude (multiple-choice questions 1–2), (**C**) attitude (Likert-scale questions 3–5), and (**D**) skill showing improvement (positive slopes), no change (horizontal lines), or deterioration (negative slopes). All test scores are in percentage values. A post-test score of zero represents all responses were incorrect; questions were unanswered; or a combination of incorrect responses and unanswered questions for a particular domain.

**Figure 3:**
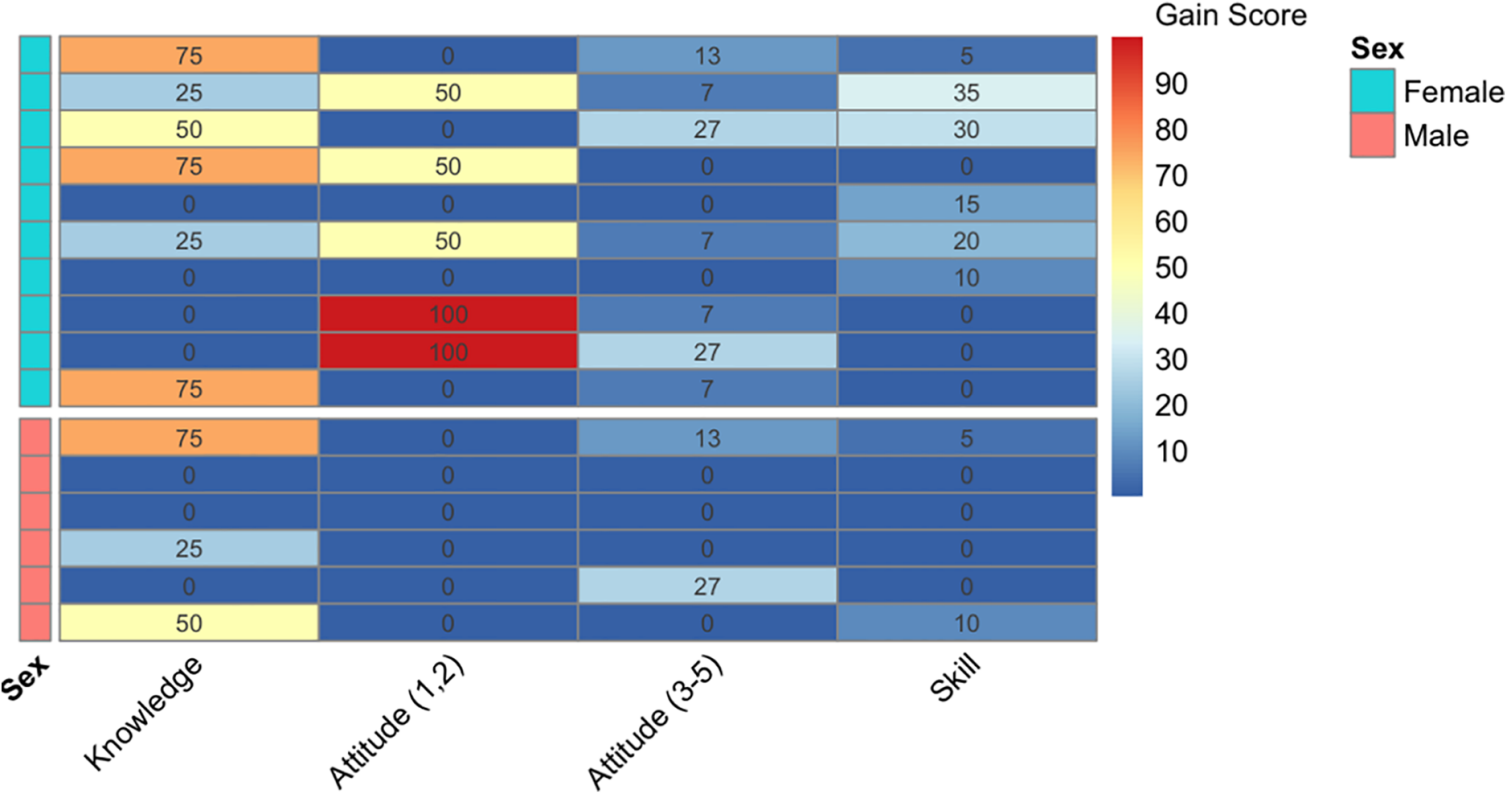
Data visualization. Mapping knowledge, attitude, and skill by sex. The magnitude of individual absolute gain scores (Δi = Gi/maximum score achievable) were illustrated on a color spectrum between blue to red, indicating low to high magnitudes. Each row represents absolute gain scores of a single participant, across learning domains. A negative or no change in gain score were treated as zero. The upper half displays female-specific scores, whereas the lower half displays male-specific scores.

Information regarding each participant's individual actual and absolute gains for knowledge, attitude, and skill assessment (*n* = 16) can be accessed in Supplementary S2. Extensive variability was observed in individual pre-test and post-test scores for persons with TBI and caregivers across learning domains, which was especially highlighted when stratified by age (Figure 2). Further, the magnitude of individual absolute gain scores was investigated relative to each learning domain and illustrated a tendency for females to experience more gains across knowledge, attitude, and skill (Figure 3).

An examination of the post-educational session evaluation revealed six of the seven individuals (86%) who attended the passive and active educational intervention reported strongly agree or agree on the first four elements. One participant reported either neutral or agree across the first four elements. With respect to the fifth element, six of the seven participants (86%) indicated no on the question; the remaining participant reported yes; however, when elaborated on the chosen answer described perceived gender bias in the context of the presented case studies and not within the educational session itself (Supplementary S1**)**.

#### Sensitivity analysis

Two participants (004, male, person with TBI, control group; 017, male, caregiver, active group) intentionally left questions unanswered on the knowledge and/or skill assessment of the post-test questionnaire due to personal beliefs. The missing values were treated as MNAR and were assigned a value of zero. Given this, we additionally conducted a sensitivity analysis on missing data to ensure that MNAR analyses did not lead to biased effectiveness estimates for the active and control groups, as compared to the passive group. For the missing values of the two participants, we inserted the most frequent value for the knowledge domain and the mean value imputation for the skill domain, where each missing value was replaced with an imputed value equal to the mean of the observed data across the three groups. The results showed an increase in individuals' post-test scores (i.e., higher than zero) and an increase in the median (IQR) post-test score of the active group for knowledge and skill respectively (i.e., 50% (IQR = 50.00%) to 75% (IQR = 50.00%); 60% (IQR = 30.00%) to 67% (IQR = 25.00%)). No difference in the median post-test score was observed for the knowledge domain of the control group. Further, individual actual and absolute gains (Supplementary S3) and group absolute, relative, and average normalized gain for the active and control groups were not impacted, given imputed values either demonstrated no gain between pre- and post-test scores or a “negative gain”.

#### Qualitative data

Two categories were identified from the attitude assessment's open-ended responses: gendered self-expectations post-injury and implications of gender stereotypes in rehabilitation, which included the need for rehabilitation treatment to look beyond sex and gender.

##### Gendered self-expectations post-injury

The first category involved unmet gendered expectations which was highlighted by individuals with TBI. Specifically, the societal and often stereotypical gendered expectations placed on oneself to embody and resume pre-injury identities and responsibilities. To illustrate this, one participant indicated: “We can place these social biases on each other through life experience and environments daily. Before the injuries, and post-TBI often we continue to place these “identities” within ourselves and have difficulty trying to be the people we once were” (007, Male, Person with TBI). Similarly, a participant emphasised that attempting to resume pre-injury roles may instead hinder recovery and noted that “Part of recovery may be embracing new roles” (001, Female, Person with TBI). A female participant additionally shared her perspective on gendered identities and roles and said the following “People will make assumptions/have expectations that may not be feasible post-injury. It took me a long time before I could make a meal for my family” (002, Female, Person with TBI). Women's gendered expectations were further highlighted by a second participant, who said:

Women's traditional gender roles are not often seen to require as much mental stimulation and so they may be asked to continue to fulfill their traditional roles while suffering from a brain injury or may feel that they are required to continue these traditional roles when suffering from a brain injury. (012, Female, Person with TBI)

##### Implications of gender stereotypes in rehabilitation

The second category involved perceived inability to receive appropriate treatment due to preconceived beliefs or gender stereotypes. To illustrate this, participants said, “If we don't fall into a stereotype, recovery can be more difficult as we may not receive appropriate treatment” (008, Female, Person with TBI) and “These stereotypes often set goals for people to get back to their normal and be able to fulfil the stereotypic requirements from the society” (014, Male, Caregiver). A female participant with TBI additionally discussed not being heard and being perceived as emotional/exaggerating during clinical decisions, resulting in insufficient rehabilitation care to promote her community participation. Further, a participant voiced that “Women tend to be seen as the weaker sex and the exercise given will reflect it” (015, Female, Person with TBI). Participants expressed the extent to which gender stereotypes and preconceived beliefs can hinder treatment and reinforce unsuitable recommendations and spoke of the need for acceptance, open-mindedness, and equity. For instance, one participant supporting a person with TBI said the following, respectively:

Meeting and understanding the injured person with as much openness as possible will allow for a treatment suitable to healing. Shoe fitting anyone into a stereotypical box is not rehabilitation. It causes further injury and impediments to recovery. Acceptance and encouragement set a safe place for rehabilitation and healing. (016, Female, Caregiver)

Further, the *need for rehabilitation treatment to look beyond sex and gender* was a subcategory identified from the perception of two research participants and concerned the preference for an individualized person-centered approach to rehabilitation that considers the different priorities of each person seeking care. For example, a participant said the following “Recovery is most effective when individuals are all treated as the individual that they are, no matter their sex or gender identification” (010, Male, Person with TBI). The second participant reiterated a similar thought and said:

I believe the person's individuality should be worked with not their sex or gender. If you think people of a certain sex or gender should be treated the same way then that blanket therapy will not identify the individuals requirements and their recovery will be hampered. (017, Male, Caregiver)

## Discussion

Existing educational interventions in TBI do not consider sex and gender in the context of brain injury medicine and rehabilitation, despite the fact that sex and gender influence people's daily experiences and a change in capacity after TBI can alter how men and women and their caregivers navigate the already difficult road of recovery. To our knowledge, this is the first pilot randomized control-group pre-test/post-test study to investigate the effectiveness of a one-time educational intervention on the knowledge, attitude, and skill of sex and gender topics in TBI, for persons with TBI and caregivers.

Findings from this study suggest that a passive educational intervention may be an effective approach. Although results for the active group did not yield meaningful learning gains, it should be noted that the same content between the passive and active groups was presented, with the marked difference being their mode of delivery (i.e., active group was interactive). Moreover, two out of five persons in the active group did not attend the intervention, as compared to one in the passive group, though these participants' responses were used in the analyses. This finding is inconsistent with previous work in which interactive education yielded better learning outcomes than passive or lecture-based teachings (52, 53), but these educational interventions were held in-person and targeted students in health-related fields, and not patients with a disability. Interestingly, a recent study investigating an online educational intervention for medical students revealed no difference between the active and passive groups in terms of learning gains and reported that students preferred information to be shared in a passive lecture-based format, instead of uncovering the information themselves in an active manner (54). This finding can potentially be relevant to patients with TBI, who may find an interactive component to require more effort and would prefer a more passive teaching style. Although results suggest that a passive educational intervention on sex and gender impacts in TBI may be effective, a larger interventional program would need to substantiate results.

The unmet gendered expectations and undue pressure that persons with TBI experience to return to pre-injury gender identities and roles were demonstrated in our qualitative findings and aligns with previous work on patients with TBI and caregivers feeling unprepared to deal with post-injury changes and losses in valued abilities and roles once discharged home (55). The inability to do gender post-TBI can therefore profoundly impact recovery and long-term outcomes (56) and should be addressed in rehabilitation without reinforcing undue gender stereotypes (57, 58).

An individualized approach to care that considers the person's unique needs and prioritizes their concerns beyond sex and gender was also expressed in the study's findings. This idea overlaps with the concept of person-centered care (59), which prioritizes the individual and their environment in the rehabilitation care approach, involves a holistic focus with the goal of preserving a meaningful life for the person (60) and individualizes dimensions of nonmedical issues and biopsychosocial relations (60). Person-centered care is recognized in healthcare delivery but remains challenging to operationalize (61). Further, part of this approach in neurorehabilitation requires the realization of patients’ new realities and the ability to target essential adaptation practices throughout rehabilitation (62). As such, sex- and gender-sensitive care (63, 64) may be incorporated within the person-centered care paradigm.

We acknowledge the limitations of our study. First, we did not reach our target sample size as recruitment challenges were experienced during the COVID-19 pandemic (30), which limited our approaches to statistical analysis. Given heterogeneity in our outcome variables across and within learning domains, we could not perform non-parametric tests (e.g., Kruskal-Wallis H test). Therefore, we presented our results descriptively, and in a visual format to ease interpretation. Second, in addition to being underpowered, three study participants did not attend the educational session on the day, though accommodations to account for their personal and work schedules were made. Third, triangulation of the qualitative component of the study would have enhanced the credibility of the findings. Fourth, there was an unequal gender representation across groups. Despite our efforts to capture educational gains across genders, we were unsuccessful in recruiting individuals who identified themselves as non-binary. Fifth, the median age for the control group (i.e., 60 years) varied greatly in comparison to that of the active and passive groups (i.e., 42 years for both), which could contribute to differences observed in gains across groups. Finally, the majority of participants were recruited from the community while in a chronic phase of disability and it was not feasible to obtain their clinical records to confirm injury severity. As such we were not able to assess the influence of injury severity in our study.

## Conclusion

We found that one session of an educational intervention on the effects of sex and gender in TBI has the potential to create knowledge, attitude, and skill learning gains among individuals with TBI and caregivers. The educational intervention can potentially raise awareness of sex and gender implications for patients with TBI and their caregivers and be beneficial in targeting relevant areas that can be raised to health care providers, including gendered expectations post-injury, and adaptations that are needed to help with recovery. Future emphasis on the effectiveness and practical implications of education on sex and gender effects in TBI can serve to provide sex- and gender-based adaptation practices in rehabilitation settings that can help persons with TBI and caregivers assimilate. Exploring the long-term effects of the education can determine its impact on relationships and recovery process.

## Data Availability

The datasets presented in this article are not readily available because we are bound by ethics at the University Health Network to not share participants’ data. Requests to access the datasets should be directed to reb@uhnresearch.ca.
