# Supplementary material for: An educational intervention to promote a culture of gender equity among persons with traumatic brain injury and caregivers: A pilot study
Source: Front Rehabil Sci. 2023 Apr 26;4:1160850. doi: 10.3389/fresc.2023.1160850 (PMC10169667; doi:10.3389/fresc.2023.1160850)
Supplement: Supplementary file 1 [file Datasheet1.docx]

**S1 Text:** Persons with TBI and Caregivers (A) Pre-test and (B) Post-test Educational Questionnaires.

1. **Pre-test Educational Questionnaire**

Date(s): ______________

**Please indicate your status** (patient with traumatic brain injury **or** significant other/caregiver)

_____________________

**How many years have you been living with a traumatic brain injury** (if you are a patient) **or supporting a patient with a traumatic brain injury** (if you are a significant other/caregiver)?

- < 1 years
- 1-5 years
- >5-10 years
- 10+ years

**Please indicate your age** (in years)

_____________________

**Please indicate your sex** (i.e., male, female, intersex)

_____________________

**Please indicate your gender** (i.e., man, woman, gender-diverse (i.e., non-binary) person)

_____________________

**KNOWLEDGE**

1. Sex and gender terms have **the same meaning**:
   1. Yes
   2. No
   3. Unsure
2. Which of the following definitions of **gender is correct***?*
3. Gender refers to our social roles, behaviours, expressions, and identities
4. Gender indicates whether an individual is sexually attracted to a member of the same or opposite sex
5. The term gender is used to describe individuals whose gender identity differs from the sex assigned at birth
6. Which of the following statements concerning **gender equality is correct**?
7. Different strategies may be required to ensure men, women and gender-diverse people with traumatic brain injury actively participate in physical rehabilitation
8. The concept of gender equality does not apply to sex-related variables such as thickness of the skull in patients after traumatic brain injury
9. Gender equality is about making rehabilitation of men, women and gender-diverse people with traumatic brain injury the same
10. **Are the expectations** towards themselves and the opposite sex the same for women, men, and gender-diverse people with traumatic brain injury?
11. Yes
12. No
13. I am not sure

If no, how do you think women, men, and gender-diverse people differ in these expectations?

_______________________________________________________________________

________________________________________________________________________________________________________________________________________________

1. What is sex (as a noun)?

________________________________________________________________________

________________________________________________________________________________________________________________________________________________

1. What is gender (as a noun)?

________________________________________________________________________

________________________________________________________________________________________________________________________________________________

1. What is **gender equity**?

________________________________________________________________________

________________________________________________________________________________________________________________________________________________

**ATTITUDES**

1. Do you believe that stereotypes about **what women, men, and gender-diverse people can and should do** can impede recovery from traumatic brain injury?

*E.g., A common stereotype is women are responsible for cooking, doing laundry and taking care of children*

*A stereotype pertaining to men is to be a “bread-winner“ for family needs*

*A stereotype pertaining to gender-diverse people is to be “at greater risk of social exclusion”*

*These stereotypes are socially created.*

***Answer the above question based on your experiences before and after the injury.***

- 1. Yes, they can impede recovery
  2. No, they cannot impede recovery
  3. I am not sure

If yes, how do you think this is so?

________________________________________________________________________

________________________________________________________________________________________________________________________________________________

1. Do you believe that clinicians’ personal believes can reinforce gender stereotypes?

*E.g., A common stereotype is women over-reporting the severity of their post-* *traumatic brain injury symptoms*

*A stereotype pertaining to men may have to do with their involvement in their injury, for example, due to intoxication, high-risk behaviours*

*These stereotypes may or may not be supported by the evidence.*

***Answer the above question based on your experiences in the healthcare system.***

- 1. Yes, they can reinforce stereotypes
  2. No, they cannot reinforce stereotypes
  3. I am not sure

If yes, how do you think this is so?

________________________________________________________________________

________________________________________________________________________________________________________________________________________________

**How strongly do you agree or disagree with the following statements?**

1. Availability of sex- and gender-specific care is essential to recovery after traumatic brain injury

Strongly agree Agree Neutral Disagree Strongly disagree

1. Clinicians have a lot to consider. Sex and gender are not a priority for me

Strongly agree Agree Neutral Disagree Strongly disagree

1. Clinicians should look beyond medical needs to see if underlying family and social difficulties are interfering with recovery from traumatic brain injury

Strongly agree Agree Neutral Disagree Strongly disagree

**SKILLS**

**Perceived ability to assess own sex & gender strengths and vulnerabilities:**

Consider that a rating of **0 indicates no confidence at all** in performing a task, and a rating of **10 indicates complete confidence** in this task. On a scale of 0 to 10, how confident are you that you can:

1. Appropriately define and distinguish between sex and gender in a traumatic brain injury context. Please select one:

0 – 1 – 2 – 3 – 4 – 5 – 6 – 7 – 8 – 9 – 10

1. Provide information to clinicians so they can create a sex and gender-specific treatment plan based on your (your family member) needs. Please select one:

0 – 1 – 2 – 3 – 4 – 5 – 6 – 7 – 8 – 9 – 10

**Ability to identify sex & gender needs to seek gender-specific care**

Read the case scenario below and answer the following questions if and how opportunity to assess sex and gender has been missed here:

*Lee, 55- year old, fell down a flight of stairs at home, did not loose consciousness, and because of ongoing nausea and vomiting was taken to the nearest emergency department by a spouse. On arrival, Lee was slightly confused and had bruises on neck and arms. Lee asked for a same-sex clinician and for assessment to be private. Lee was asked if the request stems from cultural or religious traditions, and the response was negative. Only opposite-sex clinicians were on duty at the time. Staff limitations precluded compliance with the patient’s request, and the patient was informed of this limitation. Because Lee was indecisive on how to proceed, Lee’s spouse decided on Lee’s behalf to allow a clinician of opposite-sex to complete assessment. After full assessment including a CT scan, Lee was diagnosed with concussion and discharged home the same day, to follow up with family physician and take rest as needed.*

*When asked to identify any issues related to sex and gender that might have been missed in Lee, the clinician on duty reported the following: “There are no indications that sex or gender played any role here”.*

**Please answer the following questions:**

1. Do you agree with the clinician?

Strongly agree Agree Neutral Disagree Strongly disagree

1. Do you think Lee is more likely a man, a woman or a gender-diverse person? Why do you think so?
2. Man
3. Woman
4. Gender-diverse person
5. I am not sure

If man/woman/gender-diverse person, explain your reasoning:

__________________________________________________________________________________________________________________________________________________________________________________________________________________________________________

1. Do you believe that this case reflects gender-appropriate behaviour that may impact Lee’s recovery?
2. Yes
3. No
4. I am not sure

If yes/no, explain your reasoning:

__________________________________________________________________________________________________________________________________________________________________________________________________________________________________________

1. **Post-test Educational Questionnaire**

**Date:** ______________

**ID: _______________**

**Please indicate your status** (patient with traumatic brain injury **or** significant other/caregiver)

_____________________

**How many years have you been living with a traumatic brain injury** (if you are a patient) **or supporting a patient with a traumatic brain injury** (if you are a significant other/caregiver)?

- < 1 years
- 1-5 years
- >5-10 years
- 10+ years

**Please indicate your age** (in years)

_____________________

**Please indicate your sex** (i.e., male, female, intersex)

_____________________

**Please indicate your gender** (i.e., man, woman, gender-diverse (i.e., non-binary) person)

_____________________

**EDUCATIONAL SESSION EVALUATION**

**Please rate the following aspects of the session:**

|  | **1** | **2** | **3** | **4** | **5** |
| --- | --- | --- | --- | --- | --- |
|  | Strongly Disagree | Disagree | Neutral | Agree | Strongly Agree |
| The session met the stated learning objectives | | | | | |
| Objective 1: What is the difference between sex and gender? Why is sex/gender equity important? |  |  |  |  |  |
| Objective 2: What is traumatic brain injury (TBI)? How does sex and gender affect recovery? |  |  |  |  |  |
| Objective 3: How does sex and gender affect your care? How to adjust your care plans |  |  |  |  |  |
| The overall program content … | | | | | |
| Met my expectations |  |  |  |  |  |
| Was well organized |  |  |  |  |  |
| I will use the information I learned in my life |  |  |  |  |  |

**Did you perceive any degree of unfairness towards men, women, or gender-diverse people in any part of the session?**

- YES
- NO

If YES, please describe:

**KNOWLEDGE**

1. Sex and gender terms have **the same meaning**:
   1. Yes
   2. No
   3. Unsure
2. Which of the following definitions of **gender is correct***?*
3. Gender refers to our social roles, behaviours, expressions, and identities
4. Gender indicates whether an individual is sexually attracted to a member of the same or opposite sex
5. The term gender is used to describe individuals whose gender identity differs from the sex assigned at birth
6. Which of the following statements concerning **gender equality is correct**?
7. Different strategies may be required to ensure men, women and gender-diverse people with traumatic brain injury actively participate in physical rehabilitation
8. The concept of gender equality does not apply to sex-related variables such as thickness of the skull in patients after traumatic brain injury
9. Gender equality is about making rehabilitation of men, women and gender-diverse people with traumatic brain injury the same
10. **Are the expectations** towards themselves and the opposite sex the same for women, men, and gender-diverse people with traumatic brain injury?
11. Yes
12. No
13. I am not sure

If no, how do you think women, men, and gender-diverse people differ in these expectations?

_______________________________________________________________________

________________________________________________________________________________________________________________________________________________

1. What is sex (as a noun)?

________________________________________________________________________

________________________________________________________________________________________________________________________________________________

1. What is gender (as a noun)?

________________________________________________________________________

________________________________________________________________________________________________________________________________________________

1. What is **gender equity**?

________________________________________________________________________

________________________________________________________________________________________________________________________________________________

**ATTITUDES**

1. Do you believe that stereotypes about **what women, men, and gender-diverse people can and should do** can impede recovery from traumatic brain injury?

*E.g., A common stereotype is women are responsible for cooking, doing laundry and taking care of children*

*A stereotype pertaining to men is to be a “bread-winner“ for family needs*

*A stereotype pertaining to gender-diverse people is to be “at greater risk of social exclusion”*

*These stereotypes are socially created.*

***Answer the above question based on your experiences before and after the injury.***

1. Yes, they can impede recovery
2. No, they cannot impede recovery
3. I am not sure

If yes, how do you think this is so?

________________________________________________________________________

________________________________________________________________________________________________________________________________________________

1. Do you believe that clinicians’ personal believes can reinforce gender stereotypes?

*E.g., A common stereotype is women over-reporting the severity of their post-* *traumatic brain injury symptoms*

*A stereotype pertaining to men may have to do with their involvement in their injury, for example, due to intoxication, high-risk behaviours*

*These stereotypes may or may not be supported by the evidence.*

***Answer the above question based on your experiences in the healthcare system.***

1. Yes, they can reinforce stereotypes
2. No, they cannot reinforce stereotypes
3. I am not sure

If yes, how do you think this is so?

________________________________________________________________________

________________________________________________________________________________________________________________________________________________

**How strongly do you agree or disagree with the following statements?**

1. Availability of sex- and gender-specific care is essential to recovery after traumatic brain injury

Strongly agree Agree Neutral Disagree Strongly disagree

1. Clinicians have a lot to consider. Sex and gender are not a priority for me

Strongly agree Agree Neutral Disagree Strongly disagree

1. Clinicians should look beyond my medical needs to see if underlying family and social difficulties are interfering with recovery from traumatic brain injury

Strongly agree Agree Neutral Disagree Strongly disagree

**SKILLS**

**Perceived ability to assess own sex & gender strengths and vulnerabilities:**

Consider that a rating of **0 indicates no confidence at all** in performing a task, and a rating of **10 indicates complete confidence** in this task. On a scale of 0 to 10, how confident are you that you can:

1. Appropriately define and distinguish between sex and gender in a traumatic brain injury context. Please select one:

0 – 1 – 2 – 3 – 4 – 5 – 6 – 7 – 8 – 9 – 10

1. Provide information to clinicians so they can create a sex and gender-specific treatment plan based on your (your family member) needs. Please select one:

0 – 1 – 2 – 3 – 4 – 5 – 6 – 7 – 8 – 9 – 10

**Ability to identify sex & gender needs to seek gender-specific care**

Read the case scenario below and answer the following questions if and how opportunity to assess sex and gender has been missed here:

*Lee, 55- year old, fell down a flight of stairs at home, did not loose consciousness, and because of ongoing nausea and vomiting was taken to the nearest emergency department by a spouse. On arrival, Lee was slightly confused and had bruises on neck and arms. Lee asked for a same-sex clinician and for assessment to be private. Lee was asked if the request stems from cultural or religious traditions, and the response was negative. Only opposite-sex clinicians were on duty at the time. Staff limitations precluded compliance with the patient’s request, and the patient was informed of this limitation. Because Lee was indecisive on how to proceed, Lee’s spouse decided on Lee’s behalf to allow a clinician of opposite-sex to complete assessment. After full assessment including a CT scan, Lee was diagnosed with concussion and discharged home the same day, to follow up with family physician and take rest as needed.*

*When asked to identify any issues related to sex and gender that might have been missed in Lee, the clinician on duty reported the following: “There are no indications that sex or gender played any role here”.*

**Please answer the following questions:**

1. Do you agree with the clinician?

Strongly agree Agree Neutral Disagree Strongly disagree

1. Do you think Lee is more likely a man, a woman or a gender-diverse person? Why do you think so?
2. Man
3. Woman
4. Gender-diverse person
5. I am not sure

If man/woman/gender-diverse person, explain your reasoning:

__________________________________________________________________________________________________________________________________________________________________________________________________________________________________________

1. Do you believe that this case reflects Lee’s gender-appropriate behaviour that may impact Lee’s recovery?
2. Yes
3. No
4. I am not sure

If yes/no, explain your reasoning:

_________________________________________________________________________________________________________________________________________________________________________________________________________________________________________

**S2 Table:** Individual Actual and Absolute Gains for (A) Knowledge, (B) Attitude (questions one and two and three to five) and (C) Skill.

| 1. **Knowledge** | | | | | | | | | | | | | | |
| --- | --- | --- | --- | --- | --- | --- | --- | --- | --- | --- | --- | --- | --- | --- |
| ID | Sex | Age  (yrs) | TSI | Pre-intervention Survey Questions | | | | Post-intervention Survey Questions | | | | | Individual Actual Gain (G_i_) | Individual Absolute Gain (Δ_i_) |
|  |  |  |  | **#1** | **# 2** | **# 3** | **#4** | **#1** | **#2** | **#3** | | **#4** |  |  |
| **Passive Group** | | | | | | | | | | | | | | |
| 6PT | Female | 55 | ≤5 | + | - | - | - (unsure) | + | + | + | | + | 3 | 75% |
| 7PT | Male | 42 | ≤5 | + | - | - | - (unsure) | + | + | + | | + | 3 | 75% |
| 8PT | Female | 42 | ≤5 | + | + | + | - (unsure) | + | + | + | | + | 1 | 25% |
| 16CG | Female | 75 | ≤5 | - (unsure) | + | + | - | + | + | + | | + | 2 | 50% |
| 9 PT* | Female | 27 | ≤5 | - | - | + | - (unsure) | + | + | + | | + | 3 | 75% |
| **Active Group** | | | | | | | | | | | | | | |
| 10PT | Male | 79 | ≤5 | **+** | **+** | **+** | **+** | + | + | - | | - (unsure) | 0 | 0% |
| 13PT | Female | 37 | >5 | **+** | **+** | **+** | **+** | + | + | + | | + | 0 | 0% |
| 17CG | Male | 57 | ≤5 | + | + | + | - | + | + | N/A (†) | | - | 0 | 0% |
| 11PT* | Male | 42 | ≤5 | - | - | - | - (unsure) | - | - | + | | - (unsure) | 1 | 25% |
| 12PT* | Female | 31 | ≤5 | + | + | + | - | + | + | + | | + | 1 | 25% |
| **Control Group** | | | | | | | | | | | | | | |
| 1PT | Female | 63 | ≤5 | + | - | + | + | + | - | + | + | | 0 | 0% |
| 2PT | Female | 57 | >5 | **+** | **+** | **+** | **+** | + | + | + | - (unsure) | | 0 | 0% |
| 4PT | Male | 78 | ≤5 | - | + | - | - (unsure) | - | N/A (†) | - | - (unsure) | | 0 | 0% |
| 5PT | Female | 43 | ≤5 | - | - | + | + | + | + | - | - | | 0 | 0% |
| 14CG | Male | 30 | ≤5 | + | - | + | - | + | + | + | + | | 2 | 50% |
| 15CG | Female | 71 | >5 | - | - | - | - | - (unsure) | - | + | + | | 3 | 75% |

*Non-adherent to intervention

†Did not respond to question due to personal beliefs

Abbreviations: CG – Caregiver; PT – Patient; TSI – Time Since Injury; Yrs – Years

| 1. **Attitude** | | | | | | | | | | |
| --- | --- | --- | --- | --- | --- | --- | --- | --- | --- | --- |
| ID | Sex | Age  (yrs) | TSI | Pre-intervention Survey Questions | | Post-intervention Survey Questions | | Individual Actual Gain (G_i_) | Individual Absolute Gain (Δ_i_) | |
|  |  |  |  | **#1** | **#2** | **#1** | **#2** |  |  |  |
| **Passive Group** | | | | | | | | | | |
| 6PT | Female | 55 | ≤5 | **+** | **+** | + | + | 0 | 0% | |
| 7PT | Male | 42 | ≤5 | + | - (unsure) | + | - (unsure) | 0 | 0% | |
| 8PT | Female | 42 | ≤5 | - (unsure) | + | + | + | 1 | 50% | |
| 16CG | Female | 75 | ≤5 | **+** | **+** | + | + | 0 | 0% | |
| 9 PT* | Female | 27 | ≤5 | - | + | + | + | 1 | 50% | |
| **Active Group** | | | | | | | | | | |
| 10PT | Male | 79 | ≤5 | **+** | **+** | + | + | 0 | | 0% |
| 13PT | Female | 37 | >5 | **+** | **+** | + | + | 0 | | 0% |
| 17CG | Male | 57 | ≤5 | **+** | **+** | + | + | 0 | | 0% |
| 11PT* | Male | 42 | ≤5 | - (unsure) | - (unsure) | - (unsure) | - (unsure) | 0 | | 0% |
| 12PT* | Female | 31 | ≤5 | + | - (unsure) | + | + | 1 | | 50% |
| **Control Group** | | | | | | | | | | |
| 1PT | Female | 63 | ≤5 | **+** | **+** | + | + | 0 | | 0% |
| 2PT | Female | 57 | >5 | - (unsure) | - (unsure) | + | + | 2 | | 100% |
| 4PT | Male | 78 | ≤5 | - | + | - | - (unsure) | 0 | | 0% |
| 5PT | Female | 43 | ≤5 | - | - (unsure) | + | + | 2 | | 100% |
| 14CG | Male | 30 | ≤5 | **+** | **+** | + | - | 0 | | 0% |
| 15CG | Female | 71 | >5 | **+** | **+** | + | + | 0 | | 0% |

*Non-adherent to intervention

Abbreviations: CG – Caregiver; PT – Patient; TSI – Time Since Injury; Yrs – Years

| 1. **Attitude** | | | | | | | | | | | | | |
| --- | --- | --- | --- | --- | --- | --- | --- | --- | --- | --- | --- | --- | --- |
| ID | Sex | Age  (yrs) | TSI | Pre-intervention Survey Questions | | | Post-intervention Survey Questions | | | | | Individual Actual Gain (G_i_) | Individual Absolute Gain (Δ_i_) |
|  |  |  |  | **#3** | **#4** | **#5** | **#3** | **#4** | | **#5** | |  |  |
| **Passive Group** | | | | | | | | | | | |  |  |
| 6PT | Female | 55 | ≤5 | 2 (80) | 1 (20) | 1 (100) | 1 (100) | 3 (60) | | 2 (80) | | 40 | 13% |
| 7PT | Male | 42 | ≤5 | 2 (80) | 5 (100) | 2 (80) | 1 (100) | 5 (100) | | 1 (100) | | 40 | 13% |
| 8PT | Female | 42 | ≤5 | 2 (80) | 3 (60) | 1 (100) | 1 (100) | 4 (80) | | 2 (80) | | 20 | 7% |
| 16CG | Female | 75 | ≤5 | 2 (80) | 2 (40) | 1 (100) | 1 (100) | 5 (100) | | 1 (100) | | 80 | 27% |
| 9 PT* | Female | 27 | ≤5 | 5 (20) | 5 (100) | 2 (80) | 4 (40) | 2 (40) | | 2 (80) | | 0 | 0% |
| **Active Group** | | | | | | | | | | | |  |  |
| 10PT | Male | 79 | ≤5 | 2 (80) | 4 (80) | 2 (80) | 3 (60) | | 4 (80) | | 1 (100) | 0 | 0% |
| 13PT | Female | 37 | >5 | 1 (100) | 5 (100) | 1 (100) | 2 (80) | | 5 (100) | | 1 (100) | 0 | 0% |
| 17CG | Male | 57 | ≤5 | 1 (100) | 5 (100) | 1 (100) | 5 (20) | | 2 (40) | | 2 (80) | 0 | 0% |
| 11PT* | Male | 42 | ≤5 | 1 (100) | 2 (40) | 1 (100) | 3 (60) | | 1 (20) | | 2 (80) | 0 | 0% |
| 12PT* | Female | 31 | ≤5 | 1 (100) | 3 (60) | 1 (100) | 1 (100) | | 4 (80) | | 1 (100) | 20 | 7% |
| **Control Group** | | | | | | | | | | | |  |  |
| 1PT | Female | 63 | ≤5 | 2 (80) | 2 (40) | 2 (80) | 2 (80) | 1 (20) | | 2 (80) | | 0 | 0% |
| 2PT | Female | 57 | >5 | 2 (80) | 3 (60) | 2 (80) | 2 (80) | 4 (80) | | 2 (80) | | 20 | 7% |
| 4PT | Male | 78 | ≤5 | 5 (20) | 1 (20) | 3 (60) | 4 (40) | 2 (40) | | 1 (100) | | 80 | 27% |
| 5PT | Female | 43 | ≤5 | 3 (60) | 2 (40) | 1 (100) | 2 (80) | 5 (100) | | 1 (100) | | 80 | 27% |
| 14CG | Male | 30 | ≤5 | 3 (60) | 4 (80) | 1 (100) | 3 (60) | 4 (80) | | 2 (80) | | 0 | 0% |
| 15CG | Female | 71 | >5 | 2 (80) | 4 (80) | 1 (100) | 2 (80) | 5 (100) | | 1 (100) | | 20 | 7% |

*Non-adherent to intervention

Abbreviations: CG – Caregiver; PT – Patient; TSI – Time Since Injury; Yrs – Years

| 1. **Skill** | | | | | | | | | |
| --- | --- | --- | --- | --- | --- | --- | --- | --- | --- |
| ID | Sex | Age  (yrs) | TSI | Pre-intervention Survey Questions | | Post-intervention Survey Questions | | Individual Actual Gain (G_i_) | Individual Absolute Gain (Δ_i_) |
|  |  |  |  | **A** | **B** | **A** | **B** |  |  |
| **Passive Group** | | | | | | | | | |
| 6PT | Female | 55 | ≤5 | 7 (70) | 8 (80) | 8 (80) | 8 (80) | 10 | 5% |
| 7PT | Male | 42 | ≤5 | 8 (80) | 8 (80) | 9 (90) | 8 (80) | 10 | 5% |
| 8PT | Female | 42 | ≤5 | 3 (30) | 6 (60) | 7 (70) | 9 (90) | 70 | 35% |
| 16CG | Female | 75 | ≤5 | 5 (50) | 7 (70) | 9 (90) | 9 (90) | 60 | 30% |
| 9 PT* | Female | 27 | ≤5 | 5 (50) | 5 (50) | 4 (40) | 6 (60) | 0 | 0% |
| **Active Group** | | | | | | | | | |
| 10PT | Male | 79 | ≤5 | 7 (70) | 7 (70) | 6 (60) | 5 (50) | 0 | 0% |
| 13PT | Female | 37 | >5 | 6 (60) | 8 (80) | 9 (90) | 8 (80) | 30 | 15% |
| 17CG | Male | 57 | ≤5 | 10 (100) | 10 (100) | N/A (†) | N/A (†) | 0 | 0% |
| 11PT* | Male | 42 | ≤5 | 9 (90) | 9 (90) | 6 (60) | 6 (60) | 0 | 0% |
| 12PT* | Female | 31 | ≤5 | 7 (70) | 7 (70) | 9 (90) | 9 (90) | 40 | 20% |
| **Control Group** | | | | | | | | | |
| 1PT | Female | 63 | ≤5 | 5 (50) | 2 (20) | 5 (50) | 4 (40) | 20 | 10% |
| 2PT | Female | 57 | >5 | 8 (80) | 8 (80) | 7 (70) | 7 (70) | 0 | 0% |
| 4PT | Male | 78 | ≤5 | 7 (70) | 4 (40) | 6 (60) | 0 (0%) | 0 | 0% |
| 5PT | Female | 43 | ≤5 | 7 (70) | 7 (70) | 5 (50) | 5 (50) | 0 | 0% |
| 14CG | Male | 30 | ≤5 | 6 (60) | 3 (30) | 4 (40) | 7 (70) | 20 | 10% |
| 15CG | Female | 71 | >5 | 6 (60) | 10 (100) | 7 (70) | 8 (80) | 0 | 0% |

*Non-adherent to intervention

†Did not respond to question due to personal beliefs

Abbreviations: CG – Caregiver; PT – Patient; TSI – Time Since Injury; Yrs – Years

**S3 Table:** Individual Actual and Absolute Gains for (A) Knowledge and (B) Skill Following Sensitivity Analysis.

| **A. Knowledge** | | | | | | | | | | | | | | |
| --- | --- | --- | --- | --- | --- | --- | --- | --- | --- | --- | --- | --- | --- | --- |
| ID | Sex | Age  (yrs) | TSI | Pre-intervention Survey Questions | | | | Post-intervention Survey Questions | | | | | Individual Actual Gain (G_i_) | Individual Absolute Gain (Δ_i_) |
|  |  |  |  | **#1** | **# 2** | **# 3** | **#4** | **#1** | **#2** | **#3** | | **#4** |  |  |
| **Passive Group** | | | | | | | | | | | | | | |
| 6PT | Female | 55 | ≤5 | + | - | - | - (unsure) | + | + | + | | + | 3 | 75% |
| 7PT | Male | 42 | ≤5 | + | - | - | - (unsure) | + | + | + | | + | 3 | 75% |
| 8PT | Female | 42 | ≤5 | + | + | + | - (unsure) | + | + | + | | + | 1 | 25% |
| 16CG | Female | 75 | ≤5 | - (unsure) | + | + | - | + | + | + | | + | 2 | 50% |
| 9 PT* | Female | 27 | ≤5 | - | - | + | - (unsure) | + | + | + | | + | 3 | 75% |
| **Active Group** | | | | | | | | | | | | | | |
| 10PT | Male | 79 | ≤5 | **+** | **+** | **+** | **+** | + | + | - | | - (unsure) | 0 | 0% |
| 13PT | Female | 37 | >5 | **+** | **+** | **+** | **+** | + | + | + | | + | 0 | 0% |
| 17CG | Male | 57 | ≤5 | + | + | + | - | + | + | + (†) | | - | 0 | 0% |
| 11PT* | Male | 42 | ≤5 | - | - | - | - (unsure) | - | - | + | | - (unsure) | 1 | 25% |
| 12PT* | Female | 31 | ≤5 | + | + | + | - | + | + | + | | + | 1 | 25% |
| **Control Group** | | | | | | | | | | | | | | |
| 1PT | Female | 63 | ≤5 | + | - | + | + | + | - | + | + | | 0 | 0% |
| 2PT | Female | 57 | >5 | **+** | **+** | **+** | **+** | + | + | + | - (unsure) | | 0 | 0% |
| 4PT | Male | 78 | ≤5 | - | + | - | - (unsure) | - | + (†) | - | - (unsure) | | 0 | 0% |
| 5PT | Female | 43 | ≤5 | - | - | + | + | + | + | - | - | | 0 | 0% |
| 14CG | Male | 30 | ≤5 | + | - | + | - | + | + | + | + | | 2 | 50% |
| 15CG | Female | 71 | >5 | - | - | - | - | - (unsure) | - | + | + | | 3 | 75% |

*Non-adherent to intervention

†Imputed value

Abbreviations: CG – Caregiver; PT – Patient; TSI – Time Since Injury; Yrs – Years

| **B. Skill** | | | | | | | | | |
| --- | --- | --- | --- | --- | --- | --- | --- | --- | --- |
| ID | Sex | Age  (yrs) | TSI | Pre-intervention Survey Questions | | Post-intervention Survey Questions | | Individual Actual Gain (G_i_) | Individual Absolute Gain (Δ_i_) |
|  |  |  |  | **A** | **B** | **A** | **B** |  |  |
| **Passive Group** | | | | | | | | | |
| 6PT | Female | 55 | ≤5 | 7 (70) | 8 (80) | 8 (80) | 8 (80) | 10 | 5% |
| 7PT | Male | 42 | ≤5 | 8 (80) | 8 (80) | 9 (90) | 8 (80) | 10 | 5% |
| 8PT | Female | 42 | ≤5 | 3 (30) | 6 (60) | 7 (70) | 9 (90) | 70 | 35% |
| 16CG | Female | 75 | ≤5 | 5 (50) | 7 (70) | 9 (90) | 9 (90) | 60 | 30% |
| 9 PT* | Female | 27 | ≤5 | 5 (50) | 5 (50) | 4 (40) | 6 (60) | 0 | 0% |
| **Active Group** | | | | | | | | | |
| 10PT | Male | 79 | ≤5 | 7 (70) | 7 (70) | 6 (60) | 5 (50) | 0 | 0% |
| 13PT | Female | 37 | >5 | 6 (60) | 8 (80) | 9 (90) | 8 (80) | 30 | 15% |
| 17CG | Male | 57 | ≤5 | 10 (100) | 10 (100) | (67.3) (†) | (66.0) (†) | 0 | 0% |
| 11PT* | Male | 42 | ≤5 | 9 (90) | 9 (90) | 6 (60) | 6 (60) | 0 | 0% |
| 12PT* | Female | 31 | ≤5 | 7 (70) | 7 (70) | 9 (90) | 9 (90) | 40 | 20% |
| **Control Group** | | | | | | | | | |
| 1PT | Female | 63 | ≤5 | 5 (50) | 2 (20) | 5 (50) | 4 (40) | 20 | 10% |
| 2PT | Female | 57 | >5 | 8 (80) | 8 (80) | 7 (70) | 7 (70) | 0 | 0% |
| 4PT | Male | 78 | ≤5 | 7 (70) | 4 (40) | 6 (60) | 0 (0%) | 0 | 0% |
| 5PT | Female | 43 | ≤5 | 7 (70) | 7 (70) | 5 (50) | 5 (50) | 0 | 0% |
| 14CG | Male | 30 | ≤5 | 6 (60) | 3 (30) | 4 (40) | 7 (70) | 20 | 10% |
| 15CG | Female | 71 | >5 | 6 (60) | 10 (100) | 7 (70) | 8 (80) | 0 | 0% |

*Non-adherent to intervention

†Imputed value

Abbreviations: CG – Caregiver; PT – Patient; TSI – Time Since Injury; Yrs – Years
